# Acute Kidney Injury Following Revascularization in Patients With Chronic Limb-Threatening Ischemia and Non-Dialysis-Dependent Chronic Kidney Disease: Insights From the NSQIP Database at 30-Day Follow-Up

**DOI:** 10.1177/15266028231173297

**Published:** 2023-05-16

**Authors:** Konstantinos Stavroulakis, Nikolaos Tsilimparis, Athanasios Saratzis, Barbara Rantner, Jan Stana, Anand Dayama, Mark G. Davies, Ryan Gouveia e Melo

**Affiliations:** 1 Department of Vascular Surgery, Ludwig Maximilian University Hospital, Munich, Germany; 2 Department of Cardiovascular Sciences, University of Leicester, Leicester, UK; 3 Division of Vascular and Endovascular Surgery, The University of Texas Health Science Center at San Antonio, San Antonio, TX, USA

**Keywords:** kidney outcomes, endovascular revascularization, open revascularization, chronic limb-threatening ischemia, NSQIP

## Abstract

**Background:**

Patients with chronic limb-threatening ischemia (CLTI) and chronic kidney disease (CKD) are at risk of developing renal injury following revascularization. We aimed to compare the risk of adverse renal events following endovascular revascularization (ER) or open surgery (OS) in patients with CLTI and CKD.

**Methods:**

A retrospective analysis of the National Surgical Quality Improvement Program (NSQIP) databases (2011–2017) was performed including patients with CLTI and non-dialysis-dependent CKD, comparing ER to OS. The primary outcome was a composite of postprocedural kidney injury or failure within 30 days. Thirty-day mortality, major adverse cardiac and cerebrovascular events (MACCE), amputation, readmission or target lesion revascularization (TLR) were compared using multivariate logistic regression and propensity-score matched analysis.

**Results:**

A total of 5009 patients were included (ER: 2361; OS: 3409). The risk for the composite primary outcome was comparable between groups (odds ratio [OR]: 0.78, 95% confidence interval (CI): 0.53-1.17) as for kidney injury (n=54, OR: 0.97, 95% CI: 0.39-1.19) or failure (n=55, OR: 0.68, 95% CI: 0.39-1.19). In the adjusted regression, a significant benefit was observed with ER for the primary outcome (OR: 0.60, p=0.018) and renal failure (OR: 0.50, p=0.025), but not for renal injury (OR: 0.76, p=0.34). Lower rates of MACCE, TLR, and readmissions were observed after ER. Thirty-day mortality and major amputation rates did not differ. In the propensity score analysis, revascularization strategy was not associated with renal injury or failure.

**Conclusions:**

In this cohort, the incidence of renal events within 30 days of revascularization in CLTI was low and comparable between ER and OR.

**Clinical Impact:**

In a cohort of 5009 patients with chronic limb-threatening ischemia and non-end-stage chronic kidney disease (CKD), postprocedural kidney injury or failure within 30 days was comparable between patients submitted to open or endovascular revascularization (ER). Lower rates of major adverse cardiac and cerebrovascular events, target lesion revascularization, and readmissions were observed after endovascular revascularization. Based on these findings, ER should not be avoided due to fear of worsening renal function in CKD patients with chronic limb-threatening ischemia. In fact, these patients benefit more from ER regarding cardiovascular outcomes with no increased risk of kidney injury.

## Introduction

Peripheral artery disease (PAD) is estimated to affect more than 200 million individuals globally and is associated with a considerable risk for cardiovascular morbidity and limb loss.^1^ In patients with chronic limb-threatening ischemia (CLTI), the most severe form of lower limb PAD, the cumulative incidence of mortality and major amputation has been shown to be as high as 28% at 1 year after initial presentation.^2–4^ Most patients who develop CLTI require limb revascularization to salvage the limb and/or save their life.^2^ The minimally invasive nature of endovascular revascularization (ER) for patients with CLTI might offer a benefit for these high-risk patients, who are often frail and have several co-morbidities. Currently, percutaneous endovascular procedures are considered a first-line treatment strategy for certain patients with CLTI.^4,5^

On the contrary, the performance of open and ER in specific CLTI subgroups require a separate evaluation. For instance, the treatment of patients with non-dialysis-dependent chronic kidney disease (CKD) and CLTI can be particularly challenging as they are both at risk for limb-loss and progression of their kidney disease. In fact, acute kidney injury (AKI) has been reported as being one of the most common complications of both open and ER in the context of CLTI.^5^ Previous reports suggested a benefit from ER in terms of mortality and major postoperative complications; however, the impact of the revascularization on postoperative and medium-term renal function has not been fully evaluated, especially in large series, adjusted for co-morbidities.^6^ The administration of nephrotoxic contrast agents during endovascular procedures and consequently the risk for contrast-induced nephropathy has been historically considered harmful for patients with CKD.^7,8^ Nevertheless, blood loss, the hypovolemic status of the patient as well as the requirement for general anesthesia during open surgery might impact greatly on renal function. However, little is known about the comparative risk for AKI and renal failure after revascularization. The aim of this study is to report peri-operative renal outcomes in patients with non-end-stage CKD and CLTI after surgical and ER using the American College of Surgeons National Surgical Quality Improvement Program (NSQIP) database.^9^

## Materials and Methods

This study followed the reporting guidelines from the STROBE (Strengthening the Reporting of Observational Studies in Epidemiology) statement for cohort studies.^10^

### Study Design, Setting, and Participants

This is a retrospective analysis of prospectively collected data from the procedure targeted lower extremity open (LEO) and endovascular (LEE) NSQIP database, collected between 2011 and 2017.

The targeted study population included patients with CLTI (Rutherford class 4, 5, and 6) and non-end-stage CKD. Exclusion criteria were patients with AKI at the time of surgery, end-stage renal disease requiring renal replacement therapy, and estimated glomerular filtration rate (eGFR) ≥60 mL/min/1.73 m^2^.

Asymptomatic patients or those presenting only with claudication (Rutherford class 1–3) were excluded. Patients were divided in two groups according to the type of procedure in either ER or open surgery (OS). The study was performed in line with the requirements of the local ethics committee and adhering to the Declaration of Helsinki. No ethical approval was necessary for this analysis of routinely collected fully anonymized data, based on local policies and NSQIP guidance.

### Variables, Data Sources/Measurement, Bias, and Study Size

The NSQIP database is a prospectively collected, nationally validated, risk-adjusted, outcomes-based program to measure and improve the quality of surgical care, designed to collect standardized data from more than 500 hospitals internationally.^9^

National Surgical Quality Improvement Program collects data on more than 150 variables, including patient demographics, height, weight, risk factors, functional health status, American Society of Anesthesiologist (ASA) physical status classification, previous interventions, chronic lower limb ischemia, symptoms, type of presentation, surgical details including type of surgery, duration of surgery, and type of anesthesia. Postoperative outcomes include length of stay, postoperative complications, such as surgical site infections, pneumonia, pulmonary embolism, prolonged ventilation (>48 h), kidney complications, stroke, myocardial infarction, postoperative bleeding, deep venous thrombosis, sepsis, reintervention (related and nonrelated), and readmission (related and nonrelated). Regarding patients undergoing lower limb revascularization, additional postoperative outcomes include loss of patency and major amputation. To ensure the quality of data, the data collection at each site is performed prospectively by a trained and certified surgical clinical reviewer and are subject to an interrater reliability audit.^9^

Preoperative variables collected in the targeted LEO and LEE data sets were symptomatology (which we divided into CLTI versus non-CLTI) and procedure performed (which was divided as ER or OS and further into above the knee and below the knee procedures). The 30-day-specific postoperative variables collected were renal injury, renal failure, 30-day mortality, myocardial infarction, cardiac arrest, stroke, major amputation, target lesion revascularization (TLR), related reintervention, and related readmission.

### Study Outcomes and Definitions

The primary outcome measure consisted of a composite of renal injury, defined by the NSQIP as “reduced capacity of the kidney to perform its function as evidenced by a rise in creatinine of >2 mg/dl from preoperative value, but with no requirement for dialysis within 30 days of the operation” and renal failure, defined by the NSQIP as “a patient who did not require dialysis preoperatively, developing worsening of renal function postoperatively, requiring hemodialysis, peritoneal dialysis, hemofiltration, hemodiafiltration, or ultrafiltration.”

Secondary outcome measures included: renal injury, renal failure, 30-day mortality, major adverse cardiac and cerebrovascular events (MACCEs), major amputation, TLR, reintervention related to the primary intervention and re-admission related to the primary intervention.

Open surgery included common-/deep-femoral endarterectomy, femoropopliteal bypass grafting, distal origin femoropopliteal bypass and popliteal-distal bypass. The ER included patients treated by femoropopliteal or tibial angioplasty/stenting/atherectomy. For the purposes of the subgroup analysis, we defined below-the-knee (BTK) procedures as any femoral-distal bypass or popliteal distal bypass, or the use of isolated tibial ER. Estimated glomerular filtration rate was calculated based on the 2021 Chronic Kidney Disease Epidemiology Collaboration (CKD-EPI) equation based on serum creatinine.^11^ Non-dialysis-dependent CKD was classified as a glomerular filtration rate (GFR) below 60 mL/min/1.73m^2^, but ≥15 mL/min/1.73m^2^ and not on renal replacement therapy prior to surgery.^11^

Major adverse cardiac and cerebrovascular event was defined as any of the following: 30-day all-cause mortality, myocardial infarction, cardiac arrest, or stroke. Target lesion revascularization was considered a “major re-intervention of the treated arterial segment.” A major amputation was defined as any above-ankle amputation.

### Statistical Analysis

Statistical analysis was carried out using STATA version 16.1 (Statistics/Data analysis, StataCorp LLC, Texas, USA). Continuous variables are presented as mean ± standard deviation when normally distributed and median (with interquartile range [IQR]) when not. Categorical variables are expressed in numbers (percentage). Students *t*-test was used when comparing continuous variables and the χ^2^ test to compare categorical variables; however, when appropriate, the Mann-Whitney or Fisher Exact test were used instead, respectively. Statistics are presented comparing the ER with the OS group.

A multivariate logistic regression in a forward stepwise fashion, including variables with p<0.20 on univariate analysis or those that are known risk factors, was undertaken. This analysis was performed comparing open versus endovascular surgery patients, which were further divided in above- (ATK) versus below-the-knee cohorts. Results were presented as odds ratio (OR) with 95% confidence interval (CI).

Propensity score matching (PSM) was performed to compare outcomes following ER versus OS in an adjusted manner. Variables included in the PSM process were age, sex, smoking, chronic obstructive pulmonary disease (COPD), hypertension, cancer, wound infection, bleeding disorders, type of anesthesia, previous surgery, race, diabetes, functional health status, ventilator dependency prior to surgery, chronic heart failure, hematocrit level, platelet count, steroid use, weight loss, preoperative sepsis, urgent operation, American Society of Anesthesiology class, statin medication, acetylsalicylic acid use, below or above the knee revascularization procedures, obesity (body mass index [BMI]>30); PSM was performed in a nonparsimonious fashion with a 1:1 ratio and a caliper of 0.1. All analyses were considered statistically significant if a two-tailed p value <0.05 was observed.

### Additional Analysis

All outcomes were further analyzed in the subgroup of patients with ATK and BTK revascularization. In addition, an analysis was carried out regarding possible associated factors with the composite outcome of renal injury or failure besides the method of revascularization.

## Results

### Patients, Demographics, and Risk Factors

**Figure 1.**
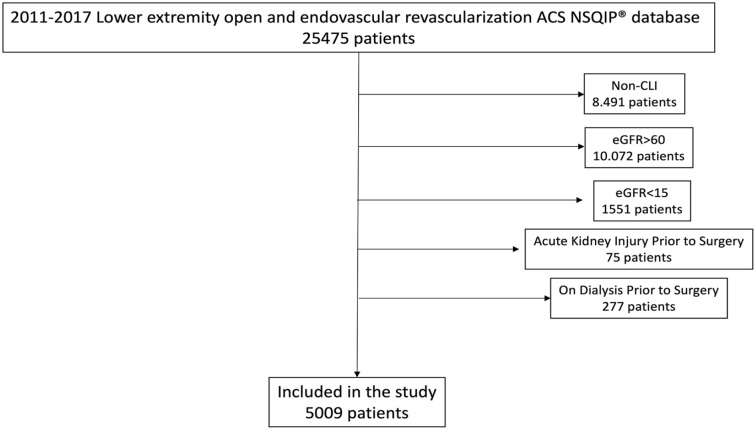
Study inclusion and exclusion diagram detailing the cohort of patients selected (study population) from the lower extremity American College of Surgeons National Surgical Quality Improvement Program (ACS-NSQIP) Non-CLI, Non Chronic Limb-threatening Ischemia..

The LEE and LEO NSQIP enrolled 25 475 patients treated between 2011 and 2017. The current analysis included 5009 patients, 2079 in the ER group and 3409 in the OS group (Figure 1). Detailed procedures are described in Supplementary Table 1. The mean age was 74 (±11), and 52.9% (n=2650) of patients were male. In the ER group, patients were older (p<0.001) and more often female (p<0.001), had a lower pre-operative eGFR (p<0.001), a higher prevalence of diabetes (p<0.001), bleeding disorders (p<0.001) and steroid drug use (p=0.030), worse functional health status (p<0.001), lower baseline hemoglobin (p<0.001) and a higher rate of pre-operative sepsis (p=0.042). Patients treated by OS had a higher prevalence of COPD (p=0.005), active smoking (p<0.001), and previous revascularizations (p<0.001). These differences were similar in both the ATK and BTK groups. Table 1 summarizes patient’s demographics and baseline characteristics.

**Table 1.** Demographics and Risk Factors.

Demographic/risk factor	All (n=5009)				Above-the-knee (n=2969)				Below-the-knee (n=2040)			
	All (n, %)	Endo(%) (n=2079)	Open (%) (n=2930)	p	All (n, %)	Endo (n, %) (n=1479)	Open (n, %) (n=1490)	p	All (n, %)	Endo (n, %) (n=600)	Open (n, %) (n=1440)	p
Age (mean, SD)	**74 (11)**	**75 (11)**	**73 (10)**	**<0.001**	**74 (11)**	**74 (11)**	**73 (10)**	**<0.001**	**74 (11)**	**75 (11)**	**74 (10)**	**0.033**
Sex (male)	**2650 (52.9)**	**1027 (49.4)**	**1623 (55.4)**	**<0.001**	**1425 (48.0)**	**670 (45.3)**	**755 (51.0)**	**0.003**	1225 (60.1)	357 (59.5)	868 (60.3)	0.744
Race (black)	1037 (20.7)	420 (20.2)	617 (21.1)	0.461	582 (19.6)	277 (18.7)	305 (20.5)	0.232	445 (22.3)	143 (23.8)	312 (21.7)	0.284
Preoperative creatinine	1.55 (0.46)	1.56 (0.48)	1.54 (0.45)	0.118	1.54 (0.46)	1.54 (0.48)	1.53 (0.45)	0.417	**1.56 (0.45)**	**1.59 (0.48)**	**1.55 (0.44)**	**0.028**
Preoperative eGFR	**44.2 (10.7)**	**43.4 (11.0)**	**44.8 (10.5)**	**<0.001**	**44.1 (10.9)**	**43.5 (11.0)**	**44.7 (10.8)**	**0.002**	**44.4 (10.5)**	**43.3 (10.8)**	**44.9 (10.3)**	**0.002**
CKD stage: 3 4	4410 (88.04) 599 (12.0)	1784 (85.8) 295 (14.2)	2626 (89.6) 304 (10.4)	**<0.001**	2590 (87.2) 379 (12.8)	1268 (85.7) 211 (14.3)	1322 (88.7) 168 (11.3)	**0.015**	1820 (89.2) 220 (10.8)	516 (86.0) 84 (14.0)	1304 (95.6) 136 (9.4)	**0.003**
HTN	4545 (90.3)	1874 (90.1)	2651 (90.5)	0.690	2672 (90.0)	1327 (89.7)	1345 (90.3)	0.620	1853 (90.8)	547 (91.2)	1306 (90.7)	0.736
Diabetes	**3059 (61.1)**	**1354 (65.1)**	**1705 (58.2)**	**<0.001**	**1769 (59.6)**	**933 (63.1)**	**836 (56.1)**	**<0.001**	**1290 (63.2)**	**421 (70.2)**	**869 (60.3)**	**<0.001**
CHF	299 (6.0)	139 (6.7)	160 (5.5)	0.071	192 (6.5)	103 (7.0)	89 (6.0)	0.272	107 (5.2)	36 (6.0)	71 (4.9)	0.324
COPD	**574 (11.5)**	**207 (10.0)**	**367 (12.5)**	**0.005**	**389 (13.1)**	**160 (10.8)**	**229 (15.4)**	**<0.001**	**185 (9.1)**	**47 (7.8)**	**138 (9.6)**	**0.210**
Smoking	**1120 (22.4)**	**347 (16.7)**	**773 (26.4)**	**<0.001**	**741 (25.0)**	**289 (19.5)**	**452 (30.3)**	**<0.001**	**379 (18.6)**	**58 (9.7)**	**321 (22.3)**	**<0.001**
Bleeding disorders	**1418 (28.1)**	**704 (33.9)**	**714 (24.4)**	**<0.001**	**875 (29.5)**	**517 (35.0)**	**358 (24.0)**	**<0.001**	**543 (26.6)**	**187 (31.2)**	**356 (24.7)**	**0.003**
Disseminated cancer	19 (0.4)	10 (0.5)	9 (0.3)	0.324	14 (0.5)	8 (0,5)	6 (0.4)	0.583	5 (0.2)	2 (0.3)	3 (0.2)	0.603
Functional health status independent: Independent Partially dependent Completely dependent	4246 (85.4) 660 (13.3) 68 (1.4)	1665 (80.7) 350 (17.0) 47 (2.3)	2581 (88.6) 310 (10.6) 21 (0.7)	**<0.001**	2519 (85.5) 383 (13.0) 44 (1.5)	1195 (81.5) 236 (16.1) 36 (2.5)	1324 (89.5) 147 (9.9) 8 (0.5)	**<0.001**	1727 (85.2) 277 (13.7) 24 (1.2)	470 (79.0) 114 (19.2) 11 (1.8)	1257 (87.7) 163 (11.4) 13 (0.9)	**<0.001**
ASA risk (>3)	1617 (32.6)	698 (33.6)	919 (31.4)	0.099	937 (31.6)	469 (31.7)	468 (31.4)	0.860	**680 (33.3)**	**229 (38.2)**	**451 (31.3)**	**0.003**
Prior intervention: None Open revascularization Endovascular revascularization	3174 (63.4) 905 (18.1) 930 (18.6)	1349 (64.9) 412 (19.8) 318 (15.3)	1825 (62.3) 493 (16.8) 612 (20.9)	**<0.001**	859 (62.6) 562 (18.9) 548 (18.5)	917 (62.0) 320 (21.6) 242 (16.4)	941 (63.2) 242 (16.2) 306 (20.5)	**<0.001**	1315 (64.5) 343 (16.8) 382 (18.7)	432 (72.0) 92 (15.3) 76 (12.7)	883 (61.3) 251 (17.4) 306 (21.2)	**<0.001**
Ventilator dependency <48 h	5 (0.10)	1 (0.05)	4 (0.14)	0.329	1 (0.03)	0 (0.00)	1 (0.07)	0.319	4 (0.2)	1 (0.2)	2 (0.2)	0.846
Steroid use	**385 (7.7)**	**180 (8.7)**	**205 (7.0)**	**0.030**	225 (7.6)	119 (8.0)	106 (7.1)	0.337	**160 (7.8)**	**61 (10.2)**	**99 (6.9)**	**0.012**
>10% weight loss 6 months	54 (1.1)	26 (1.2)	4428 (1.0)	0.319	28 (0.9)	12 (0.8)	16 (1.1)	0.459	**26 (1.3)**	**14 (2.3)**	**12 (0.8)**	**0.006**
Preoperative albumin (mean, SD)	3.38 (0.65)	3.37 (0.70)	3.39 (0.64)	0.506	3.41 (0.65)	3.39 (0.66)	3.44 (0.63)	0.123	3.34 (0.66)	3.34 (0.69)	3.35 (0.64)	0.879
Preoperative hemoglobin (mean, SD)	**11.4 (2.0)**	**11.2 (1.9)**	**11.5 (2.0)**	**<0.001**	**11.4 (0.2)**	**11.3 (2.0)**	**11.5 (1.9)**	**0.001**	**11.3 (2.0)**	**11.1 (1.9)**	**11.4 (2.0)**	**0.003**
Anemic (WHO criteria)	3549 (70.8)	1474 (70.9)	2075 (70.8)	0.951	2015 (67.9)	1012 (68.4)	1003 (67.3)	0.518	1534 (75.2)	462 (77.0)	1072 (74.4)	0.223
Pre op Sepsis	**263 (5.2)**	**125 (6.0)**	**138 (4.7)**	**0.042**	146 (4.9)	78 (5.3)	68 (4.6)	0.371	117 (5.7)	47 (7.8)	70 (4.9(	0.921
BMI>30	1641 (32.8)	704 (33.9)	937 (32.0)	0.162	972 (32.7)	498 (33.7)	474 (31.8)	0.280	669 (32.8)	206 (34.3)	463 (32.1)	0.339
BMI<18.5	190 (3.8)	77 (3.7)	113 (3.9)	0.780	110 (3.7)	54 (3.65)	56 (3.76)	0.877	80 (3.9)	23 (3.8)	57 (4.0)	0.895

Abbreviations: ASA, American society of Anesthesiology; BMI, body mass index; CHF, chronic heart failure; CKD, chronic kidney disease; COPD, chronic obstructive pulmonary disease; HTN, hypertension; WHO criteria- anemia as hemoglobin <12.5 g/dl. Bold variables indicate statistically significant differences.

Tissue loss was the indication for treatment in 67% of the included patients (n=3357). Endovascular revascularization was preferred over OS in these patients (71.4% versus 63.9%, p<0.001). This difference was more pronounced in the BTK subgroup (83.2% versus 69.9%, p<0.001). Most patients underwent ER under local/regional anesthesia (77.8%, n=1774), whereas general anesthesia was preferred in patients treated by OS (93.6%, n=2744), p<0.001. The procedural time was significantly lower in the ER group compared to OS (112 ± 69 min versus 243±109 min, p<0.001). Table 2 provides an overview of the procedural details.

**Table 2.** Procedure Details.

Procedure details	All				Above the knee				Below the knee			
	All	Endo	Open	p	All	Endo	Open	p	All	Endo	Open	p
Emergency	262 (5.2)	98 (4.7)	164 (5.6)	0.166	156 (5.2)	73 (4.9)	83 (5.6)	0.438	106 (5.2)	25 (4.2)	81 (5.6)	0.176
CLTI stage 5–6 (Rutherford)	**3357 (67.0)**	**1485 (71.4)**	**1872 (63.9)**	**<0.001**	**1851 (62.3)**	**986 (66.7)**	**865 (58.1)**	**<0.001**	**1506 (73.8)**	**499 (83.2)**	**1007 (69.9)**	**<0.001**
General anesthesia	**3331 (66.5)**	**587 (28.2)**	**2744 (93.65)**	**<0.001**	**1816 (61.2)**	**433 (29.3)**	**1383 (92.8)**	**<0.001**	**1515 (74.3)**	**154 (25.7)**	**1361 (94.5)**	**<0.001**
Operation time (min)	**188 (114)**	**112 (69)**	**243 (109)**	**<0.001**	**169 (107)**	**113 (71)**	**225 (107)**	**<0.001**	**215.9 (120)**	**107 (64)**	**261 (108)**	**<0.001**

Abbreviation: CLTI, chronic limb-threatening ischemia. Bold variables indicate statistically significant differences.

Regarding the specific procedures performed (Supplemen-tary Table 1), the most common procedure was femoropopliteal angioplasty/stenting/atherectomy (n=1479, 29.5%), followed by femoropopliteal bypass with single segment saphenous vein (n=739, 14.7%), femoropopliteal bypass with prosthetic/spliced vein/composite (n=712, 14.2%), femorodistal bypass with single-segment saphenous vein (n=600, 12.9%), tibial angioplasty/stenting (n=600, 12.0%), femorodistal bypass with prosthetic/spliced vein/composite (n=412, 8.2%), popliteal distal bypass with single-segment saphenous vein (n=295, 5.9%), popliteal distal bypass with prosthetic/spliced vein/composite (n=85, 1.7%), femoral endarterectomy (n=36, 0.7%), and profundoplasty (n=3, 0.1%).

### PRIMARY OUTCOME—Composite of Renal Injury and/or Failure

No differences were found for the composite outcome of renal injury or failure after ER and OS (1.8%, n=38 versus 2.3%, n=68, OR: 0.78 (95% CI: 0.53-1.17), p=0.232; Table 3). However, the logistic regression analysis showed a lower rate for the composite outcome following ER (adjusted Odds Ratio (aOR): 0.60 (95% CI: 0.39-0.92, p=0.018; Table 4). The subgroup analysis revealed a benefit only for the BTK treatment group but not for patients treated for femoropopliteal disease (Table 4). In addition, the logistic regression revealed a comparable risk for developing the primary outcome between patients with grade 3 and grade 4 CKD. The incidence of renal injury or failure in the different CKD stages was comparable after ER and OS (Supplementary Table 2). An additional analysis identified risk factors for the composite outcome (Supplementary Table 3). The preoperative eGFR (for every decrease in 10 mL/min/1.73m²) (aOR: 0.66, 95% CI: 0.55-0.78, p<0.001), the presence of diabetes (aOR: 1.65 (95% CI: 1.02-2.66, p=0.042); chronic heart failure (aOR: 2.73, 95% CI: 1.55-4.88, p<0.001); bleeding disorders (aOR: 1.71, 95% CI: 1.14-2.57, p=0.010), and emergency surgery (aOR: 2.70, 95% CI: 1.00-4.94, p=0.001) was associated with a higher incidence of postoperative renal injury or failure (Supplementary Table 4).

**Table 3.** Univariate Analysis.

Outcomes	All					Above the knee					Below the knee				
	All	Endo	Open	OR (95% CI)	p	All	Endo	Open	OR (95% CI)	p	All	Endo	Open	OR (95% CI)	p
Renal Injury	54 (1.1)	22 (1.1)	32 (1.1)	0.97 (0.56-1.66)	0.909	31 (1.0)	17 (1.1)	14 (0.9)	1.23 (0.60-2.50)	0.574	23 (1.1)	5 (0.8)	18 (1.2)	0.66 (0.24-1.80)	0.420
Renal Failure	55 (1.1)	18 (0.9)	37 (1.3)	0.68 (0.39-1.19)	0.184	29 (1.0)	12 (0.8)	17 (1.1)	0.71 (0.34-1.49)	0.364	26 (1.3)	6 (1.0)	20 (1.4)	0.72 (0.29-1.79)	0.478
Composite acute kidney injury or failure	106 (2.1)	38 (1.8)	68 (2.3)	0.78 (0.53-1.17)	0.232	58 (1.9)	28 (1.9)	30 (2.0)	0.94 (0.56-1.58)	0.813	48 (2.3)	10 (1.7)	38 (2.6)	0.62 (0.31-1.26)	0.191
30-day mortality	142 (2.8)	60 (2.9)	82 (2.8)	1.03 (0.74-1.44)	0.854	84 (2.8)	49 (3.3)	35 (2.3)	1.42 (092-2.21)	0.115	58 (2.8)	11 (1.8)	47 (3.3)	0.55 (0.28-1.07)	0.081
MACCE	**104 (5.0)**	**104 (5.0)**	**207 (7.1)**	**0.69 (0.54-0.88)**	**0.003**	164 (5.5)	78 (5.3)	86 (5.8)	0.91 (0.66-1.24)	0.553	**147 (7.2)**	**26 (4.3)**	**121 (8.4)**	**0.49 (0.32-0.76)**	**0.001**
Major amputation	210 (4.2)	80 (3.8)	130 (4.4)	0.86 (0.65-1.14)	0.306	100 (3.4)	50 (3.4)	50 (3.4)	1.01 (0.68-1.50)	0.970	110 (5.4)	30 (5.0)	80 (5.6)	0.89 (0.58-1.38)	0.613
Target lesion revascularization	**217 (4.3)**	**76 (3.7)**	**141 (4.8)**	**0.75 (0.56-1.0)**	**0.048**	123 (4.1)	58 (3.9)	65 (4.4)	0.89 (0.62-1.28)	0.547	**94 (4.6)**	**18 (3.0)**	**76 (5.30)**	**0.55 (0.33-0.94)**	**0.027**
Re-operation related to the primary intervention	**559 (11.2)**	**142 (6.8)**	**417 (14.2)**	**0.44 (0.36-0.54)**	**<0.001**	**290 (9.8)**	**95 (6.4)**	**195 (13.1)**	**0.46 (0.25-0.59)**	**<0.001**	**269 (13.2)**	**47 (7.8)**	**222 (15.4)**	**0.47 (0.33-0.65)**	**<0.001**
Readmission related to the primary intervention	**577 (11.5)**	**167 (8.0)**	**410 (14.0)**	**0.54 (0.44-0.65)**	**<0.001**	**319 (10.7)**	**107 (7.2)**	**212 (14.2)**	**0.47 (0.37-0.60)**	**<0.001**	**258 (12.6)**	**60 (10)**	**198 (13.7)**	**0.70 (0.51-0.95)**	**0.021**

Abbreviations: CI, confidence interval; MACCE, major adverse cardiac and cerebrovascular events; OR, odds ratio. Bold variables indicate statistically significant differences.

**Table 4.** Logistic Regression.

Outcomes	All (ER versus OS)			Above the knee (ER versus OS)			Below the knee (ER versus OS)		
	aOR	95% CI	p	aOR	95% CI	p	aOR	95% CI	p
Renal Injury	0.76	0.43-1.34	0.343	0.89	0.42-1.88	0.763	0.44	0.14-1.37	0.158
Renal Failure	**0.50**	**0.27-0.92**	**0.025**	0.55	0.25-1.22	0.143	0.45	0.16-1.27	0.131
Composite of renal injury or renal failure	**0.60**	**0.39-0.92**	**0.018**	0.69	0.40-1.20	0.193	**0.44**	**0.21-0.96**	**0.038**
30-day mortality	0.80	0.56-1.15	0.226	1.15	0.71-1.85	0.571	0.37	0.18-0.77	0.008
MACCE	**0.58**	**0.45-0.75**	**<0.001**	0.76	0.55-1.07	0.117	**0.38**	**0.24-0.61**	**<0.001**
Major amputation	0.86	0.64-1.15	0.309	1.02	0.67-1.55	0.920	0.85	0.54-1.35	0.494
Target lesion revascularization	**0.72**	**0.54-0.98**	**0.035**	0.83	0.57-1.22	0.347	0.59	0.34-1.01	0.056
Reoperation related to the primary intervention	**0.43**	**0.35-0.53**	**<0.001**	**0.43**	**0.33-0.57**	**<0.001**	**0.47**	**0.33-0.66**	**<0.001**
Readmission related to the primary intervention	**0.49**	**0.40-0.60**	**<0.001**	**0.43**	**0.33-0.56**	**<0.001**	**0.62**	**0.45-0.85**	**0.004**

Abbreviations: aOR, adjusted odds ratio; CI, confidence interval; ER, endovascular revascularization; MACCE, major adverse cardiac and cerebrovascular events; OR, odds ratio; OS, open surgery.

### Secondary Outcomes

#### Renal injury

The overall rate of postoperative renal injury was 1.1% (n=54) and no significant differences were found between groups (1.1%, n=22 versus 1.1%, n=32, OR: 0.97 (95% CI: 0.39-1.19, p=0.909). The risk for AKI did not differ in the subgroups of ATK and BTK revascularizations (Table 3). In an adjusted logistic regression, the proportion of those developing renal injury remained comparable between groups (aOR: 0.76, 95% CI: 0.43-1.34, p=0.343). The logistic regression showed comparable proportions developing renal injury for patients with grade 3 (aOR: 0.92, CI: 0.49-1.73, p=0.488) and grade 4 CKD (aOR: 0.28, CI: 0.05-1.53, p=0.126).

#### Renal failure

Postprocedural renal failure was observed in 55 patients (1.1%) with no significant differences between the two groups (0.9%, n=18 versus 1.3%, n=37, OR: 0.68, 0.39-1.19, p=0.184). Following logistic regression, ER reduced the risk for renal failure (aOR: 0.50, 95% CI: 0.27-0.92, p=0.025). The treatment strategy was not associated with the incidence of renal failure in ATK and BTK disease and in patients with CKD stage 4 (Table 4); in the adjusted logistic regression, there was a lower incidence of renal failure in patients with CKD stage 3 having ER (aOR 0.47 (95% CI: 0.23-0.98, p=0.045; Supplementary Table 2).

#### Other secondary outcomes

Overall, ER was associated with less MACCEs (OR 0.69, 95% CI: 0.54-0.88, p=0.003), TLR (OR 0.75, 95% CI: 0.56-1.0, p=0.48), CLTI-related reintervention (OR 0.44, 95% CI: 0.36-0.54, p<0.001), and CLTI-related readmission (OR 0.54, 95% CI: 0.44-0.65, p<0.001). Both 30-day mortality (OR 1.03, 95% CI: 0.74-1.44, p=0.85) and major amputation rates (OR 0.86, 95% CI: 0.65-1.14, p=0.31) did not differ significantly between ER and OS. The risk for MACCE and TLR were only significantly different in the BTK group, whereas CLTI-related reintervention and readmission were significant in both ATK and BTK groups (Table 3).

In the adjusted logistic regression, the risk for MACCE (aOR: 0.58, 95% CI: 0.45-0.75, p<0.001); TLR (aOR: 0.72, 95% CI: 0.54-0.98, p=0.035); CLTI-related reinterventions (aOR: 0.43 95% CI: 0.35-0.53, p<0.001); and CLTI-related readmission (aOR:0.49, 95% CI: 0.40-0.60, p<0.001) was also lower in the ER group. Regarding the ATK and BTK subgroups, both CLTI-related reintervention and readmission remained significantly different, whereas incidence of MACCE was only different in the BTK group. On the contrary TLR, 30-day mortality, and major amputation rates were comparable after ATK and BTK procedures (Table 4).

### Propensity Score Analysis

Following PSM, 1415 patients were matched in each group (ER vs. OS). The treatment strategy did not have any influence on the composite primary outcome (OR: 0.78, 95% CI: 0.46-1.32, p=0.350) and the secondary outcomes of renal injury (OR 0.72, 95% CI: 0.35-1.47, p=0.369) or renal failure (OR: 0.86 (95% CI: 0.41-1.82), p=0.704). The postoperative occurrence of MACCE was significantly lower in the ER group (OR: 0.65, 95% CI: 0.48-0.89, p=0.007), while the 30-day mortality did not differ significantly between the two treatment strategies (OR: 0.84 (95% CI: 0.54-1.30, p=0.431; Table 5).

**Table 5.** Propensity Score Match.

Outcomes	All PSM ER (n=1415) versus OS (n=1495)		
	OR	95% CI	p
Renal Injury	0.72	0.35-1.47	0.369
Renal Failure	0.86	0.41-1.82	0.704
Composite of renal injury or renal failure	0.78	0.46-1.32	0.350
MACCE	0.65	0.48-0.89	0.007
30-day mortality	0.84	0.54-1.30	0.431

Abbreviations: CI, confidence interval; ER, endovascular revascularization; MACCE, major adverse cardiac and cerebrovascular events; OR, odds ratio; OS, open surgery; PSM, propensity score match.

## Discussion

Lower extremity atherosclerosis is a common finding amongst patients with CKD. The presence of CKD is an important parameter regarding the choice of treatment algorithms and greatly influences clinical outcomes in CLTI, regardless of revascularization strategy.^4,12–14^ Although previous studies evaluated the impact of revascularization strategy on the fate of the affected limb or mortality, the relative risk for renal events in large multicenter series including patients with CLTI has not been widely reported or examined in adjusted analyses. This is the first multicenter large study comparing the risk for postoperative renal injury or failure after endovascular and open revascularization in patients with non-dialysis-dependent CKD and CLTI. The main finding of this analysis is that ER does not increase the risk for periprocedural renal events compared to OS. Furthermore, ER was associated with a significantly lower rate of MACCE, TLR, and CLTI-related readmission compared to surgery. Both unadjusted and carefully adjusted analyses (using two different statistical approaches) were performed.

There is a general concern regarding ER in patients with non-end-stage CKD due to the risk of contrast-induced nephro-toxicity, which can lead to a worsening of renal function in the short term.^7,8,15^ Katsogridakis et al described a rate of 11.7% of AKI and 17.5% of major adverse kidney events (MAKE; death, dialysis, a reduction in eGFR at least 25%) at 30 days in patients submitted to ER of symptomatic femoropopliteal artery disease; no comparisons were made with open surgical revascularization, and the series only included three major vascular centers.^5^ Based on previous findings and assumptions surrounding the renal impact of contrast administration, endovascular procedures are often avoided in patients with both CKD and CLTI, which might impact on clinical outcomes, especially because this is a population with several co-morbidities.^5,7,8^ However, the current body of evidence suggests that ER can be offered as a first-line strategy in patients with chronic renal function impairment. Contrast-induced nephropathy has been shown to be relatively uncommon and rarely related to long-lasting renal failure with current nonionic and iso-osmolar contrast agents.^7^ In addition, individuals with CKD represent a subgroup of CLTI patients that might particularly benefit from ER.^4,6,14^ In this cohort, ER was associated with lower rates of MACCE, re-intervention and re-admission, with no significant differences after PSM for postoperative adverse kidney outcomes or 30-day mortality. These findings were also consistent in more severe (stage-4 CKD) stages of CKD (Supplementary Table 2) and in all our reported adjusted analyses.

The comparable risk for postoperative renal injury or failure after open surgery is an important parameter regarding the treatment selection algorithm in patients with CLTI and CKD.^14^ In fact, AKI following open revascularization is not uncommon. Blood loss has been considered one of the major factors leading to postoperative renal decline.^16^ Other parameters such as hypotension, increased levels of circulating catecholamines and inflammatory mediators, oxidative stress and administration of nephrotoxic agents (e.g. medication or contrast to perform a completion or diagnostic angiogram) may also contribute to renal decline after open revascularization.^17,18^

Moreover, certain baseline characteristics were found to have a much higher impact on postoperative renal outcomes than the type of revascularization, such as preoperative eGFR value, diabetes, chronic heart failure, bleeding disorders, and the emergency of the repair (Supplementary Table 4). Therefore, ER should not be excluded based only on the preoperative renal function alone, and several factors should be taken into consideration in clinical decision-making algorithms for these patients. Finally, in very high-risk patients for contrast-induced nephropathy, the use of CO2-based angiography and intravascular ultrasound might further reduce the need for the nephrotoxic agent.^2,19^ This, however, has yet to be proven in randomized trials.

In this context, the peri-operative prevention of renal decline requires meticulous risk factor modification^5,16,20^ as preoperative, intraoperative, and postoperative factors all play a role in the development of this pathology.^20^ Discontinuation of nephrotoxic agents, avoidance of hypotension, perioperative statin use, preoperative hydration using balanced crystalloids, and adequate glycemic control have all been shown to have a potential benefit and are crucial for the perioperative management of these patients.^5,20-22^

### Limitations

This study is not without limitations. Although the NSQIP database includes a high number of patients, allowing a robust matching and adjustment methods, this study carries the well-known limitations of registries and data collected in this manner. In addition, only 30-day outcomes are reported, and the long-term effects of the revascularization strategy cannot be reported. Given the limitations of the NSQIP dataset, we do not have access to precise urine output measurements or daily serum creatinine values postoperatively (i.e. we cannot report rates of AKI or rates of MAKE in a systematic and validated manner). At the same time, the NSQIP provides careful reports on renal injury and failure rates, albeit, using their own definitions, as used in this series. The lack of postoperative serum creatinine values also limits our analysis regarding the precise magnitude of kidney injury and how quickly it might have recovered. Further, contrast agent volume and blood loss were not available in the dataset, limiting the understanding of possible mechanisms related to renal decline. Additionally, the rates of renal injury in the database are lower than what has been described in other studies, which may imply some reporting bias. However, since the aim of the study is to compare renal outcomes of ER and OS, this effect may not significantly influence our outcomes. Future prospective multicenter studies are still required to allow a clear understanding of the mechanisms and implications of MAKE following lower limb revascularization in patients with CKD.

## Conclusion

This large observational study has shown that ER was not associated with a higher risk for post-operative renal injury or failure compared to open surgery in patients with CLTI and non-end stage CKD. Endovascular revascularization was associated with lower rates of MACCE, TLR, and CLTI related readmission and re-intervention. Future studies should evaluate the long-term impact of the selected treatment strategy on kidney function.

## Supplemental Material

sj-docx-1-jet-10.1177_15266028231173297 – Supplemental material for Acute Kidney Injury Following Revascularization in Patients With Chronic Limb-Threatening Ischemia and Non-Dialysis-Dependent Chronic Kidney Disease: Insights From the NSQIP Database at 30-Day Follow-Up Supplemental material, sj-docx-1-jet-10.1177_15266028231173297 for Acute Kidney Injury Following Revascularization in Patients With Chronic Limb-Threatening Ischemia and Non-Dialysis-Dependent Chronic Kidney Disease: Insights From the NSQIP Database at 30-Day Follow-Up by Konstantinos Stavroulakis, Nikolaos Tsilimparis, Athanasios Saratzis, Barbara Rantner, Jan Stana, Anand Dayama, Mark G. Davies and Ryan Gouveia e Melo in Journal of Endovascular Therapy
