# Supplementary material for: Acute Kidney Injury Following Revascularization in Patients With Chronic Limb-Threatening Ischemia and Non-Dialysis-Dependent Chronic Kidney Disease: Insights From the NSQIP Database at 30-Day Follow-Up
Source: J Endovasc Ther. 2023 May 16;32(1):214–24. doi: 10.1177/15266028231173297 (PMC11707963; doi:10.1177/15266028231173297)
Supplement: sj-docx-1-jet-10.1177_15266028231173297 – Supplemental material for Acute Kidney Injury Following Revascularization in Patients With Chronic Limb-Threatening Ischemia and Non-Dialysis-Dependent Chronic Kidney Disease: Insights From the NSQIP Database at 30-Day Follow-Up [file sj-docx-1-jet-10.1177_15266028231173297.docx]

**Supplementary table 1. Procedure details**

| **Procedure** | n | % |
| --- | --- | --- |
| Femoral Endarterectomy | 36 | 0.72 |
| Profundoplasty | 3 | 0.06 |
| Femoropopliteal bypass with single segment saphenous vein | 739 | 14.75 |
| Femoropopliteal bypass with prosthetic/spliced vein/composite | 712 | 14.21 |
| Femorodistal bypass with single segment saphenous vein | 648 | 12.94 |
| Femorodistal bypass with prosthetic/spliced vein/composite | 412 | 8.23 |
| Popliteal distal bypass with single segment saphenous vein | 295 | 5.89 |
| Popliteal distal bypass with prosthetic/spliced vein/composite | 85 | 1.70 |
| Femoropopliteal angioplasty/stenting/atherectomy | 1479 | 29.53 |
| Tibial angioplasty/stenting | 600 | 11.98 |

**Supplementary table 2. Logistic Regression according to subgroups of chronic kidney disease**

| **Procedure details** | **All (Endo versus OR)** | | | **Above-the-knee (Endo versus OR)** | | | **Below-the-knee (Endo versus OR)** | | |
| --- | --- | --- | --- | --- | --- | --- | --- | --- | --- |
|  | aOR | 95% CI | p | aOR | 95% CI | p | aOR | 95% CI | p |
| Renal Injury   - Grade 3 - Grade 4 | 0.92 | 0.49-1.73 | 0.488 | 1.04 | 0.44-2.44 | 0.934 | 0.59 | 0.18-1.92 | 0.385 |
|  | 0.28 | 0.05-1.53 | 0.126 | 0.01 | 0.0-0.92 | 0.046 | - | - | - |
| Renal Replacement Therapy   - Grade 3 - Grade 4 | 0.47 | 0.23-0.98 | 0.045 | 0.51 | 0.20-1.28 | 0.153 | 0.40 | 0.10-1.50 | 0.173 |
|  | 0.49 | 0.14-1.62 | 0.242 | 1.03 | 0.14-7.59 | 0.977 | 0.53 | 0.06-4.30 | 0.549 |
| Renal Injury or Renal replacement therapy   - Grade 3 - Grade 4 | 0.66 | 0.41-1.08 | 0.408 | 0.74 | 0.39-1.39 | 0.350 | 0.49 | 0.20-1.19 | 0.116 |
|  | 0.41 | 0.16-1.04 | 0.16 | 0.50 | 0.12-2.13 | 0.349 | 0.30 | 0.05-1.72 | 0.178 |

**Supplementary table 3. Univariate analysis for the composite outcome**

| **Demographic/ Risk Factor** | **Composite renal injury or renal failure** | |
| --- | --- | --- |
|  | OR (95%CI) | p |
| Age (every 10 year increase) | 0.82 (0.69-0.98) | 0.030 |
| Sex (male) | 1.08 (0.73-1.59) | 0.706 |
| Race (black) | 1.12 (0.71-1.78) | 0.619 |
| **Pre-operative Creatinine** | **2.38 (1.75-3.23)** | **<0.001** |
| **Pre-operative eGFR (every 10 increase)** | **0.64 (0.54-0.76)** | **<0.001** |
| CKD stage (3a as comparator):   - 3a - 3b - 4 | - 3b: 1.20 (0.76-1.88) - 4: 2.94 (1.81-4.78) | 0.430  **0.001** |
| HTN | 1.03 (0.53-1.98) | 0.936 |
| **Diabetes** | **1.99 (1.27-3.10)** | **0.003** |
| **CHF** | **3.13 (1.84-5.33)** | **<0.001** |
| COPD | 0.62 (0.30-1.29) | 0.205 |
| Smoking | 0.61 (0.36-1.04) | 0.072 |
| Wound infection | 1.38 (0.93-2.05) | 0.105 |
| **Bleeding disorders** | **1.75 (1.18-2.59)** | **0.005** |
| **ASA Risk (>3)** | **1.56 (1.06-2.31)** | **0.025** |
| Prior intervention (compared to none):   - None - Open revascularization - Endovascular revascularization | 1.03 (0.61-1.72)  1.16 (0.71-1.89) | 0.923  0.555 |
| **Ventilator dependency <48h** | **11.44 (1.32-98.86)** | **0.027** |
| Steroid use | 1.70 (0.94-3.07) | 0.077 |
| >10% weight loss 6 months | 0.87 (0.12-6.36) | 0.892 |
| **Pre-op albumin** | **0.52 (0.37-0.73)** | **<0.001** |
| Pre op platelets | 1.0 (1.0-1.0) | 0.817 |
| **Pre op HTC** | **0.95 (0.92-0.99)** | **0.008** |
| **Pre op Sepsis** | **2.84 (1.59-5.06)** | **<0.001** |
| **BMI>30** | **1.53 (1.03-2.26)** | **0.033** |
| **Emergency** | **3.11 (1.77-5.44)** | **<0.001** |
| CLI stage 5-6 (Rutherford) (compared to stage 5) | 1.19 (0.78-1.82) | 0.409 |
| General Anesthesia | 1.34 (0.87-2.07) | 0.177 |
| **Operation Time (for every increase in 10 min)** | **1.02 (1.0-1.04)** | **0.005** |
| Below the knee | 1.21 (0.82-1.78) | 0.335 |
| Endovascular surgery | 0.78 (0.52-1.17) | 0.233 |

**Supplementary table 4. Logistic regression for the composite outcome**

| **Demographic/ Risk Factor** | **Composite Renal Injury or Renal Failure** | |
| --- | --- | --- |
|  | aOR (95%CI) | p |
| Age (every 10 year increase) | 0.91 (0.74-1.11) | 0.333 |
| Sex (male) | 1.08 (0.72-1.61) | 0.701 |
| Race (black) | 1.22 (0.75-1.97) | 0.425 |
| **Pre-operative GFR (for every increase of 10)** | **0.66 (0.55- 0.78)** | **<0.001** |
| HTN | 0.81 (0.41-1.60) | 0.546 |
| **Diabetes** | **1.65 (1.02-2.66)** | **0.042** |
| **CHF** | **2.73 (1.55-4.80)** | **<0.001** |
| COPD | 0.58 (0.27-1.23) | 0.155 |
| Smoking | 0.62 (0.34-1.11) | 0.107 |
| **Bleeding disorders** | **1.71 (1.14-2.57)** | **0.010** |
| ASA Risk (>3) | 1.21 (0.80-1.83) | 0.234 |
| Ventilator dependency <48h | 4.27 (0.39-46.79) | 0.415 |
| Steroid use | 1.69 (0.92-3.12) | 0.091 |
| Pre op Sepsis | 1.85 (0.99-3.47) | 0.054 |
| BMI>30 | 1.23 (0.80-1.88) | 0.339 |
| **Emergency** | **2.70 (1.47-4.94)** | **0.001** |
| Operation Time (for every increase in 10 min) | 1.02 (1.0-1.04) | 0.058 |
| Endovascular surgery | 0.81 (0.48-1.34) | 0.430 |
